# Beyond a Single Story: Understanding Cultural Invalidations, Colorism, and Their Impact on Belongingness Among Black College Students

**DOI:** 10.3390/bs16020298

**Published:** 2026-02-19

**Authors:** Jasric J. Bland, Alexandrea R. Golden, Asya T. Miles, Myahkia X. Watson, Júlia F. M. Soares, India J. Montague, Stacy E. Herard

**Affiliations:** 1 Department of Psychology, University of Memphis, Memphis, TN 38152, USA; 2 Department of Psychology, Oklahoma State University, Stillwater, OK 74078, USA

**Keywords:** college students, Black youth, cultural invalidations, belongingness, colorism

## Abstract

Peers often serve as sources of support and protection in educational spaces. However, when cultural norms are perceived to be violated, the criticism of peers can create stressful and unwelcoming environments. Presently, little is known about the specific effect cultural invalidations (when the authenticity of one’s cultural identity is questioned by same-race peers) have on one’s identity development and sense of belonging. To address this gap, the current study qualitatively examined instances of cultural invalidations between Black undergraduate students and their same-race peers that led to feelings of inauthenticity and lack of belonging. Participants included 20 Black undergraduate students (50% female, 50% male) attending a predominantly White institution who participated in individual interviews. Reflexive thematic analysis was used to address two research questions: (a) How do Black undergraduate students perceive and interpret experiences of cultural invalidations within their Black peer groups? (b) How do cultural invalidations experienced from Black peers relate to individuals’ sense of belonging? Two themes were identified regarding participants’ experiences of cultural invalidations: (a) colorism is a source of cultural invalidation, and (b) Blackness is not a monolith. Findings also revealed the negative implications of cultural invalidations on participants’ sense of belonging. Implications for research and practice are discussed.

## 1. Introduction

Chimamanda Ngozi Adichie warned of the dangers of a single story, stating, “The single story creates stereotypes. And the problem with stereotypes is not that they are untrue, but that they are incomplete. They make one story become the only story” (TED, 2009). Historical racism against Black Americans has resulted in systems of oppression that are not only perpetuated by non-Blacks but have also impacted perspectives and interactions within the Black community. Within-group discrimination has been a silent yet significant source of division within the Black community for decades. From the complexities of skin color (i.e., structural oppression; M. Hunter, 2002; Azibo, 2014) to the invalidation of one’s Blackness due to physical traits (i.e., hair texture, eye color, and facial features) and style of speech and dress, Black individuals that fall outside of norms set by the Black community and larger society are often ostracized. While it is critical to acknowledge the impact historical underpinnings (i.e., racism) and microlevel influences (e.g., social interactions) can have on the development of one’s identity, life experiences, perspectives, and socialization (Wilder & Cain, 2011), it is equally as important to consider the spectrum of the Black experience as we challenge the narrative of a single story.

One visually salient characteristic on which Black individuals vary across the spectrum is skin tone. Colorism is defined as a form of discrimination based on skin color that often occurs within racial groups (Keyes et al., 2020). Historically, colorism has been associated with specific social and cultural meanings, including assumptions about authenticity and social worth (Keyes et al., 2020; Reece, 2018; Russell-Cole et al., 2013). These biases can often contribute to cultural or identity-based invalidations. Cultural or identity-based invalidations are interactions in which Black individuals’ experiences, aspects of identities, and cultural expressions are questioned or devalued by same-race peers (Douglass et al., 2016; Durkee et al., 2019, 2022; Franco et al., 2016). Current literature highlights the harmful effects of cultural invalidations on racial identity development and adjustment among Black youth (Durkee et al., 2022; Williams et al., 2019). Given the salient nature of race and skin tone within intraracial dynamics, cultural or identity-based invalidations, including those associated with colorism, may play a critical role in shaping the way Black emerging adults experience belonging and acceptance within their same-race peer groups.

While peers can often be a source of support and serve as protective mechanisms for individuals in educational spaces (Golden et al., 2018), research suggests that peers also contribute to harsh criticism and stress when “group-specific norms are violated” (Contrada et al., 2001; Durkee & Williams, 2015, p. 28). Given the importance of peer relationships and acceptance in emerging adulthood for navigating new levels of autonomy and identity development, the impact of within-group discrimination from peers is particularly meaningful (Douglass et al., 2016; Erikson, 1968; Savin-Williams & Berndt, 1990). These experiences can have detrimental impacts on individuals, especially during critical developmental periods when identity development and social sensitivity are particularly salient (i.e., emerging adulthood; Durkee et al., 2022). For Black college students, in particular, these peer relationships are critical as they can be seen as sources of racial affirmation, shared lived experiences, and validation within a predominantly White campus.

While the current literature provides insight into various accounts of cultural invalidations, there remains a gap in understanding how these interactions influence within-group belongingness and identity development among Black emerging adults. Taking this into account, examining the impacts of cultural invalidations in peer relationships is crucial to assess the maladaptive impacts associated with within-group alienation or perceived differences. Accordingly, the current study examines instances of cultural invalidations experienced by Black undergraduate students from Black peers and assesses the impact these encounters have on their sense of belonging.

### 1.1. Systemic Racism and the Black Community

Racism is both a belief system and an institutional structure that privileges a group that has been identified as dominant while disadvantaging those deemed as inferior, resulting in unequal access to resources and opportunities (Williams et al., 2019). The system of racism is based upon the social construction of race in which individuals can be classified as part of a caste based upon their skin tone, hair texture, and facial features (Wilkerson, 2020; Williams et al., 2019). Historically, legally sanctioned discriminatory and oppressive practices have been upheld to maintain the larger system of racism to the detriment of racially oppressed groups, not limited to Black individuals. Exposure to racism has led to disparities in the physical and mental health, education, wealth, and more of racially minoritized groups, particularly Black Americans, across the lifespan (Assari et al., 2021; Bali & Alvarez, 2004; Herring & Henderson, 2016; Williams et al., 2019). As such, scholars have focused on the impacts of racism primarily from a macro level to better understand not only the impacts of racism but also to identify factors to ameliorate its effects in the lives of Black individuals.

Racism research often focuses on the impacts of structural racism (macro level) or interpersonal racial discrimination perpetrated by the out-group. However, intragroup bias (i.e., colorism) is also a consequence of racism that occurs among Black individuals. (Alexander & Carter, 2022; Ball et al., 2023; Schmitt et al., 2014). These experiences of group-based discrimination can lead to negative outcomes regarding mental health and well-being (Ball et al., 2023).

The widespread and diffuse effects of systemic racism at the macro level permeate into lower levels of the ecological system. This means that while racism is more difficult to pinpoint, it touches every environment that individuals encounter through implemented practices and policies. It also influences the microsystem (e.g., interpersonal interactions) through the beliefs adopted by individuals as a result of cultural norms (Landor & McNeil Smith, 2019; Rosario et al., 2021; Wilder & Cain, 2011). Accordingly, intragroup bias based upon one’s skin tone, hair texture, and facial features (i.e., colorism) has been noted as a detrimental consequence of racism that remains understudied. As such, the current study addresses this gap in the literature by examining Black college students’ experiences and perceptions of cultural invalidations from same-race peers.

### 1.2. Theoretical Framework

The minority stress theory highlights the unique, ongoing stressors that ethnically and racially minoritized groups may encounter, which are rooted in broader societal structures and institutions (Ilagan et al., 2025; Meyer, 1995). Further, the theory offers a framework for understanding how chronic stressors, such as invalidations, exclusion, and identity threat, may disrupt psychological well-being and belonging among peers. Prior literature suggests identity-based or cultural invalidations function as minority stressors that negatively impact emotional regulation (Cardona et al., 2022; Ilagan et al., 2025). Cynthia García Coll et al.’s (1996) integrative model extends this perspective by emphasizing the role of race, ethnicity, gender, and social class as intersections that shape the developmental pathway of children of color. While the model was developed to highlight the experiences of children of color, factors identified within the model are relevant to the developmental processes of adolescents (Dotterer & James, 2018) and emerging adults (Hong et al., 2021; Quinn et al., 2020). Through this framework, Coll and colleagues discuss the impact that social factors, such as skin color and racial features, can have on one’s developmental process. While these social factors do not directly impact one’s development or environment, they are mediated through racism, prejudice, discrimination, and oppression. These systemically oppressive systems are combated through the creation of inhibiting and promoting environments that shape adaptive cultures. An adaptive culture is informed by a group’s collective historical and present demands and “involves a social system defined by sets of goals, values, and attitudes, that differs from the dominant culture” (Coll et al., 1996, p. 1896). For Black individuals, the development of an adaptive culture includes the consideration of the mainstream experience, the minority experience, and the Black cultural experience (Boykin & Toms, 1985; Coll et al., 1996).

For Black individuals who fall outside of the norms defined by their adaptive culture, this intended protective factor can become harmful and impact their overall sense of belonging with same-race individuals. Prior literature indicates Black youth are often ostracized by members of their own race for practicing norms considered to be aligned with White culture (Fordham & Ogbu, 1986; Durkee et al., 2019). In this way, the use of adaptive culture fails to account for diverse identities and practices within the Black community. Historically, theories centered on race and identity have failed to consider within-group differences that may impact an individual’s experience (Volpe et al., 2022). As such, the current study seeks to fill this gap by highlighting the experiences of Black individuals who face cultural invalidations most often related to behaviors, attitudes, and physical characteristics deemed as “non-Black” or “not Black enough” by the Black community.

### 1.3. Consequences of Cultural Invalidations

Cultural invalidations are defined as insults and threats to identity that are used to lessen the validity of a person’s membership within one or more social identities (Douglass et al., 2016; Durkee et al., 2019, 2022; Franco et al., 2016). While racial bias is frequently attributed to White perpetrators, ethnic-racial minority individuals often endure instances of cultural invalidation from members of the same racial group as well. Cultural invalidations within Black communities have been noted in the study of racial teasing and colorism (Douglass et al., 2016; Leath et al., 2023; Lewis & White, 2021).

#### 1.3.1. Racial Teasing

Racial teasing refers to interactions that use race as the basis for jokes or derogatory comments, often under the guise of humor. This teasing can range from subtle to overtly offensive, reinforcing societal stereotypes and prejudices (Walton et al., 2013). Furthermore, racial teasing is not limited to direct comments but can also manifest as microaggressions, stereotype threats, and racial bullying. For example, racial teasing may include mocking someone’s accent, cultural practices, or physical features, complicating the individual’s struggle with racial identity development and intensifying the negative perceptions from others (Durkee et al., 2019; Franco & Franco, 2016). Although racial teasing has significant implications for both individuals and society, research suggests that adolescents often perceive racial teasing and intragroup accusations such as “acting White” as less harmful than more historically recognized forms of discrimination (Douglass et al., 2016).

The term “acting White” refers to a racial insult that implies that one’s behaviors, such as their speech patterns, clothing style, or hobbies, are aligned with White norms (Williams et al., 2019; Durkee et al., 2019; Neal-Barnett et al., 2010). This harmful accusation can lead to detrimental psychological and overall health outcomes for individuals (Durkee & Gómez, 2021; Oh & Nicholson, 2021). When repeatedly subjected to racial jokes or derogatory comments, individuals may start to internalize these messages, believing the negative stereotypes about their racial group to be true. This process can increase symptoms of anxiety, depression, and a sense of inadequacy, as well as individuals’ self-esteem and alter how they view themselves and their place within society (Durkee & Williams, 2015; Neal-Barnett et al., 2010; Yip, 2015). For example, when examining the effects of the frequency of being accused of acting White among college students, Durkee and Gómez (2021) found that individuals who encountered this accusation more frequently during their first year of college were more likely to experience higher anxiety and depressive symptoms than individuals who had fewer encounters. Further, cultural invalidations have been linked to lower self-esteem, negative well-being, and increased suicidal ideation and attempts (Campbell & Troyer, 2007; Coleman & Carter, 2007; Franco & Franco, 2016; Lou et al., 2011; Townsend et al., 2009). Considering these significant impacts to one’s well-being and mental health, assessing the impact of cultural invalidations continues to be dire. As such, the current study will explore instances of cultural invalidations among Black peers and their impact on a lack of belongingness.

#### 1.3.2. Colorism

Colorism is defined as race-based discrimination in which those with a shared racial identity may experience “advantages and disadvantages…based on the lightness or darkness of their skin tone” and their accompanying physical characteristics (Keith & Monroe, 2016). While colorism is often understood as emerging as part of White supremacist systems, it is also important to acknowledge its historical roots in class-differentiated practices within the Black community (Frazier, 1957).

The historical implications of the perceived superiority of those with closer approximations to Whiteness and Eurocentric beauty standards have divided the Black community for centuries and created an “us versus them” mentality, in which some are viewed as “not Black enough” while others’ capabilities and physical attraction are seen as dependent on skin tone (American Psychological Association, 2018; Major et al., 2023; Coard et al., 2006). Such perspectives greatly persist in today’s society, with leadership positions and esteemed titles frequently being held by those of a lighter complexion rather than the contrary (Webb, 2021; Samuels, 2010). As such, these deeply ingrained and inescapable messages about the supremacy of lightness over darkness continue to be passed along from one generation to the next. In turn, these ideologies incessantly influence Black individuals’ formations of self-identity and social networks, leading young Black children to inevitably remain cognizant of the realities of colorism amidst their own interactions with peers of various complexions (Abrams et al., 2020).

Colorism research has primarily focused on the ways in which individuals who are of lighter skin tones experience more privilege than individuals with darker skin tones, with an emphasis on the impacts of oppression on individuals with darker skin tones. Further, studies have inarguably documented the societal implications of skin tone hierarchy on dark-skinned individuals (Keyes et al., 2020; Sissoko et al., 2023). Several studies have highlighted that individuals with darker skin tones are assumed to be more connected to the Black community and their struggles due to more exposure to racial oppression and historical narratives regarding deeper connections to a collective Black experience (M. L. Hunter, 2013). This perception potentially leads to greater acceptance within the Black community. Other research, however, indicates that skin tone alone does not predict skin tone satisfaction (Maxwell et al., 2015). Maxwell and colleagues (Maxwell et al., 2015) also found that individuals with darker skin tones reported stronger positive feelings about being Black.

The discord that is struck between Black individuals with differing skin tones can be detrimental to the well-being and development of individuals with lighter skin tones, as well as individuals with darker skin tones. For example, Oh and colleagues (Oh et al., 2021) demonstrated that in-group colorism was more detrimental to the psychiatric functioning of Black individuals than out-group colorism. This artificially constructed divide has resulted in an in-group versus out-group ideal where individuals with lighter skin tones are ostracized or pushed to the margins of their racial group based on assumed privilege due to being multi-racial or otherwise (Landor & McNeil Smith, 2019). In order to promote a strong and unified front of resistance to end racism and promote wellness among Black Americans, it is important to mend the breach within the Black community that has been perpetrated and perpetuated by systemic racism. As such, it is critical that there is a recognition and understanding of the detrimental impacts of colorism on both Black Americans with darker skin tones and Black Americans with lighter skin tones. However, little research has examined the impacts of colorism as a cultural invalidation on lighter-skinned Black Americans’ experiences and feelings of belonging within the Black community, specifically within the college environment. The current study will address this gap by examining how Black undergraduate students’ experiences and perceptions of cultural invalidations, including skin tone, impact their experiences and sense of belonging among other Black undergraduate students.

### 1.4. Present Study

Prior research demonstrates the negative roles cultural invalidations play in the lives of emerging adults, including detriments to mental and physical health and belongingness within one’s community (Campbell & Troyer, 2007; Coleman & Carter, 2007; Franco & Franco, 2016; Lou et al., 2011; Townsend et al., 2009). While this literature is extensive, the continuum of the Black experience and belongingness among peers remains understudied. As such, the present study extends this literature by centering the lived experiences of Black college students and examining how instances of cultural invalidations emerge and are interpreted within Black peer relationships. The current study included 20 Black undergraduate students with diverse life experiences. Black college students represent a critical population for understanding peer dynamics, as the college context is a particularly salient developmental period during which peer interactions play a crucial role in identity development, social belonging, and well-being (Douglass et al., 2016; Golden et al., 2018; Erikson, 1968; Savin-Williams & Berndt, 1990). By including Black undergraduates with diverse lived experiences, this study captures an in-depth exploration of how intraracial cultural invalidations are experienced and understood.

The present study was guided by the following research questions:

Research Question 1: How do Black undergraduate students perceive and interpret experiences of cultural invalidations within their Black peer groups?

Research Question 2: How do cultural invalidations experienced from Black peers relate to individuals’ sense of belonging?

## 2. Method

### 2.1. Participants

Data from the current study comes from a larger multi-method study examining peer racial socialization. Data was collected between 2017 and 2018, a notable time in which the United States was adjusting following a tumultuous presidential race that featured incendiary and divisive rhetoric. The current study included 20 self-identified Black undergraduate students who attended a Predominantly White Institution (PWI) in the southeastern region of the United States, ranging in age from 18 to 25 years (*M* = 20.61, *SD* = 2.28; n = 18). Fifty percent of the population identified as female, and the remainder identified as male. Participants ranged in classification, with 15% freshmen, 35% sophomores, 30% juniors, and 20% seniors. Sixty percent of participants indicated that their friend groups at the time of the study were majority Black, 40% indicated having a racially diverse friend group, and no participants indicated that their friend group was majority White.

### 2.2. Procedure

Following approval from the Institutional Review Board at the University of South Carolina (IRB: Pro00067334), the second author’s institution at the time of data collection, participants were recruited using a variety of methods, including campus organizations, university listservs, flyers, and snowball sampling. Interested individuals completed a brief screener to determine eligibility for participation in the study. Participants were included in the study based on their eligibility, and attention was paid to equal representation among students of male and female genders and to diverse representation of individuals’ tenure on the college campus. Participants engaged in a 30–60 min interview (*M* = 52 min, *SD* = 17.28), responding to questions such as “What are the challenges you and your friends experience being Black?” and “How does race come up when you’re with your friends?” At the end of each interview, the interviewer engaged in a member check in which themes that emerged across the interview were repeated to the participant, in addition to inquiries about accuracy and missing information. The interviews were conducted by a Black-identifying researcher. While colorism and cultural invalidations were not central to the original research study, we consider disclosure of the interviewer’s racial identity as critical, as racial identity and phenotypic traits (such as skin tone) may impact participants’ willingness to share experiences related to these relevant topics. Participants received a gift card as remuneration for their participation in the study. Pseudonyms were used in the current study to protect the confidentiality of participants.

### 2.3. Data Analysis

Six trained coders engaged in thematic analysis (Braun & Clarke, 2006) to analyze the research questions. In accordance with thematic analysis, transcripts were first read over to gain familiarity with the content. Next, initial emergent codes associated with the research questions were identified using a subset of interviews. Once initial codes were identified, coders engaged in discussion to identify larger themes from the initial codes that emerged from the data and to create a codebook. The creation of the codebook was an iterative process in which the codebook was applied to a subset of interviews and adjusted as necessary to gain a clear understanding among coders. Once the codebook was solidified, it was applied to all interviews. Coding was completed when interrater reliability reached 70%, as indicated by Cohen’s Kappa. Discrepancies in coding were discussed among the coding team until they reached a consensus.

### 2.4. Positionality

Data analysis was conducted by six self-identified women of color. This work provides an interdisciplinary perspective, as the authors have training in school psychology, clinical-community psychology, or general psychology. This includes one doctoral-level researcher, three doctoral-level graduate students, one master’s-level graduate student, and two advanced (i.e., junior and senior) undergraduate students. At the time of the study, all researchers were studying or conducting research at a Historically White Institution in the Southern region of the United States. Collectively, our positionalities uniquely situate us to conduct this work because we all study the experiences of racially minoritized individuals and have witnessed or personally encountered instances of cultural invalidations. As scholars of color who have been affected by cultural invalidations and colorism, we approached the data with awareness of how our experiences might influence interpretation. As such, we engaged in ongoing reflexive team discussions to identify possible assumptions and over-identification during the data analysis process. These practices aided in ensuring that our interpretations were consistently grounded in the participants’ narratives rather than our personal experiences. Intentional amplification of participants’ experiences required deep reflection and continued development in our understanding of the dynamic and heterogeneous nature of the Black experience. In addition to our personal connection to the work, this particular group brought an intergenerational perspective to the work that highlights the continued impact of cultural invalidations across time. Given the nature of this work, team members were encouraged to participate in practices that positively supported their well-being. The first author checked in on these practices weekly with each team member.

## 3. Results

Two themes were identified that illuminated instances of cultural invalidations from within-group peers. These themes highlight the continuum of the Black experience and the impacts of cultural invalidation on Black undergraduate students. The themes identified include: (a) colorism is a source of cultural invalidation, and (b) Blackness is not a monolith. Consistent with the second research question, we subsequently explored how these experiences related to Black undergraduate students’ sense of belonging

### 3.1. Colorism Is a Source of Cultural Invalidation

Cultural invalidations were noted through discussions of colorism, including skin tone and hair texture. Historically, skin tone has been a source of contentious division among Black individuals. While prior research has highlighted the impacts of colorism on darker-skinned individuals (Brown et al., 2023; Hall, 2017; M. Hunter, 2007), participants in the present study amplified the ways in which having lighter skin tones was associated with interactions with Black peers, including in the invalidation of one’s Black identity. Although Black individuals range greatly in skin tone, participants with lighter skin were assumed to not be Black, which created tension given the importance some participants placed on Blackness being a key aspect of their identities. For example, Jasmine describes an intense pride in being Black and the frustration that occurs when Black peers invalidate her identity based on her skin complexion.

*Well for me personally, because I don’t really look Black people just assume like I’m not Black or I’m not Black enough. […] Black history is very important to me‘cause I want people to know I am Black and I want to be Black. I don’t have any White in my family; like my whole family is Black. So, for people to see me based on my skin is kind of disappointing.*

Jasmine’s experience highlights feelings of disappointment surrounding the invalidation of her experience as a Black woman due to her lighter complexion. Further, she addresses cultural heritage as a focal point of proving her Blackness. The construct of “blackness” has been historically used to describe an assumed collective set of social experiences and specific phenotype (Brunsma & Rockquemore, 2002). However, this notion is harmful to those within the Black community, as phenotypic traits such as skin color do not neatly map onto a singular collective identity, given the variation that exists within the community itself (Brunsma & Rockquemore, 2002). This construct often complicates the individual’s struggle with racial identity and the negative perceptions from others (Durkee et al., 2019; Franco & Franco, 2016).

Other participants shared these same sentiments related to skin tone and the historical underpinnings of colorism within the Black community. Ayanna showcases the influence that colorism can have on peer interactions and the disguising of within-group discrimination as “jokes”.

*That’s another thing. I guess being in my skin tone and everything, my friends say, they joke about like, especially in the older days. You know, if you were mixed or something, you’d be a house slave. And then if you were darker, you’re out in the fields.*

Ayanna’s experience highlights viewpoints and perceptions that have been passed down through generations. Further, she describes how these generational attitudes influence the way peers view the legitimacy of her Blackness.

*They always say that I get the easier side of being Black, because I’m so light, so I can fit in with the White people if I wanted. And then being younger, when I was younger and stuff, always being around White people.*

For some participants, this lived experience goes beyond skin tone and includes multiple aspects of their identity, such as tone of voice, style of dress, and hair texture. Makayla details the challenge of protecting her intersecting identities when interacting with Black peers.

*[…] I’m half Black, I have a little bit of West Indian in me. But, like, when people ask me if I’m mixed, I sometimes want to say no. Because I feel like, there’s a stigma around, like, Black girls can’t have nice hair. So, you have to be mixed. I have a Black friend, her hair is longer than mine. Hers is almost down to her back, and she’s not mixed. And she gets it too. She’s like, people have asked her if she’s—if she’s mixed and she’ll say no. And they’re like, “well, you have to be mixed because your hair can’t be this long. Or your skin tone can’t be like this.” And I get it all the time too. And it’s just like why? And it’s mostly, sometimes it is from Black people. A lot of times.*

Makayla’s account highlights the common stereotype attached to the length of Black women’s hair. Further, her story captures the unique experience of biracial individuals that is often overlooked. The experience of individuals who are a part of multiple cultural groups is crucial, as biracial individuals may experience cultural invalidations in complex and nuanced ways. This is observed through Makayla’s hesitation and refusal to make statements that seemingly conform to this stereotype through the acknowledgment of how her multiple identities shape the ways in which she is perceived.

In totality, these instances shared by participants stress the notion that our experiences are beyond skin deep and emphasize the importance of acknowledging the spectrum of the Black experience.

### 3.2. Blackness Is Not a Monolith

Perceptions of behavior and racially ascribed actions play a significant role in shaping individuals’ experiences and identities within the Black community. The narratives provided by participants showcase the complexity and diversity within Black identity, further challenging the simplistic categorization of Blackness as a monolith. Through their experiences and interactions, participants illuminate how perceptions of “acting Black” or “acting White” are not only subjective but also do not accurately capture the breadth of Blackness. These narratives reveal the nuanced ways in which individuals negotiate their identities within a society and create an adaptive culture that often imposes strict expectations based on racial norms. Participants’ reflections highlight the internal conflicts and external pressures they face in reconciling their authentic selves with Black peer perceptions and expectations of Blackness. Their stories defy the static frameworks that seek to confine individuals within narrow definitions of race and culture. Titiana highlighted the scrutiny from peers for personal choices regarding appearance.

*Like if we’re talking about hairstyles or hair types, makeup, or just everyday things I guess […] Um, yeah, a lot of times males say, ‘why you gotta be such a White girl?’*

This quote exposes the pressure individuals face to conform to norms of Blackness (e.g., assumed style of dress, pattern of speech, hobbies), where deviation from these cultural expectations is met with accusations of “acting White” from Black peers. However, Titiana confidently asserted her individuality, refusing to adhere to prescribed adaptive norms and challenging the idea that their actions are inherently tied to their racial identity.

*So, it’s like whatever. I mean accepted or not. If that’s what you call it, I don’t feel like it’s White. I don’t feel like it’s Black. I feel like it’s just me.*

Briana reflected on the impact of language sharing with Black peers, stating, “*Like playfully, my friends, all the time, tell me that I talk White.*” This acknowledgment highlights the complexity of linguistic diversity within the Black community, where the use of language is often politicized between White English and Black English (Ogbu, 1999) and subjected to external judgment. Similarly, Marquis made a remark about their friend, highlighting their deviation from racial expectations.

*But she acts kind of, almost White and she really didn’t know, you’d think she was…she has a lot of, like, White tendencies, I guess I would say.*

This comment illuminates the diversity of cultural expression within the Black community by challenging the misconception that all Black individuals share uniform speech patterns or behaviors and also by acknowledging that participants can inadvertently be perpetuators of these cultural invalidations, too. Cultural invalidations can directly impact one’s sense of belonging. When forming one’s racial identity, there is the possibility that the authentic self will not align with peer expectations. However, this misalignment with expectations may not inherently suggest that individuals do not feel connected with their racial group. Participants discuss the tension that comes between their authentic identity and the expectations put upon them by the Black community.

Another experience shared by Makayla highlights the internalization of harmful historical cultural norms and stereotypes as she details her internal dialogue when deciding between joining a Black or White sorority.

*Like, I can blend in with Black people, but I know there’s certain things, like, I’m not really loud and I’m not the stigma Black person. But then, if I go to the White sorority, I’ll be, like, one of the only White people, I meant Black people. So, it’s just like, stuff like that I’ve had a problem with. Like, I like being Black, but then there’s obstacles that I have to go through.*

This experience depicts the complex reality of Black individuals as both the victims and perpetrators of stigmas and biased assumptions. Further, it illuminates the difficult task of oscillating between two worlds when an individual does not align with cultural norms or societal expectations of a particular group. This idea of navigating two worlds has historically been connected to Du Bois (1903) notion of double consciousness, which highlights the challenge and psychological stress of being one’s authentic self while also contending with the way societal norms often force Black individuals to see themselves through the lens of others.

The narratives provided by participants emphasize the multifaceted nature of Black identity by debunking the notion of a monolithic Black experience (Badr, 2023; Rogers, 2001). Through their experiences with perception, language, and cultural preferences, participants assert their individuality and challenge restrictive notions of racial authenticity. These insights amplify the importance of recognizing and celebrating the diversity within Blackness to advocate for a more nuanced understanding of identity that transcends simplistic racial and cultural categorizations. Further, they highlight the critical need to examine implicit biases among Black individuals.

### 3.3. Sense of Belonging

The second research question further examined the impact of these instances of peer cultural invalidations on feelings of belongingness. A firm sense of belonging is paramount during emerging adulthood, as it indicates acceptance by one’s cultural group and the ability to activate the peer group as a protective factor. The stories shared by Black undergraduate participants indicated an underlying feeling of a lack of belonging among Black peers. For some participants, these experiences also signaled intense isolation as they struggled to fit in with groups outside of their race. Participants shared encounters related to their desire to belong and the impact that ostracization can have on their journey of identity formation and authenticity. Jasmine’s account showcases this desire and details her experience with joining Black organizations as a way to prove her place within the Black community.

*I guess when I join like organizations like [NAME REDACTED] that are like mostly majority black people inside–then I show like “hey I am part your community and I have the same issues you do.”*

Jasmine’s experience highlights actions some individuals feel necessary to take to demonstrate their proximity to their Blackness to other Black peers. Other participants, such as Brandon, felt stronger connections with individuals outside of their racial group, noting a disconnect with same-race individuals.

*Because […] I felt like I had stronger connections with-like, White people versus, like, Black people. ‘Cause I couldn’t really connect to Black people.*

While Brandon and Jasmine’s experiences indicate a desire to belong with either a Black or non-Black community, some participants attributed feelings of lack of belonging to not fitting in with Black or White individuals. Ayanna exemplifies this idea by evaluating her experience in Black and White spaces.

*Like I said, I don’t fit in with the Blacks or the Whites, so being in PWI, I don’t fit in. But I also feel like if I went to a HBCU, I wouldn’t fit in either.*

Further, she described how being characterized as “preppy” influences her ability to fit in with the Black community on campus.

*I was always seen as the preppy Black kid. I don’t know, so I never really fit in ‘cause a Black girl … Here on campus, we have, you know, it’s a PWI, so it’s like you have all the White kids, and then there’s the Black community here on campus, but I feel like I don’t fit in enough with them either, so…*

Ayanna’s expressed isolation due to being her authentic self illuminates the importance of recognizing the obstacles individuals face when challenging the norms set by an adaptive culture. Similarly, Jamal details the complex duality of being perceived as “better than” by Black peers due to academics but being left out by White individuals due to his race.

*[…] I guess the like you say the White people I guess didn’t like me because I was Black, and my Black friends didn’t like me because I wasn’t in their same classes. So, I guess they thought I was like too good for them or whatever.*

Overall, reflections shared by Black participants indicated a desire to belong in Black communities and spaces on campus. However, this innate belonging is often discredited by instances of cultural invalidations. Further, these stories reflect the social consequences of deviating from cultural and societal expectations, even when these deviations are crucial components of one’s authentic self. While some participants found it important to prove their Blackness amidst these invalidations, others confidently defied stigmas and stereotypes as they related to their Black identity, even at the expense of within-group belonging.

## 4. Discussion

The current study examined how Black undergraduate students experience cultural invalidations from same-race peers and how those experiences influence their sense of belonging. We found that cultural invalidations occur among Black undergraduate students through colorism and ascriptions to beliefs that Blackness is monolithic. These invalidations resulted in participants feeling less of a sense of belonging with their Black peers. This work extends the current literature by providing a more in-depth exploration of how cultural invalidations occur among Black undergraduate students attending a PWI and how these experiences impact their sense of belonging among their same-race peers.

First, participants highlighted how colorism, through discussions and “jokes,” contributed to cultural invalidations. This was primarily noted in participants’ discussions of being judged for their lighter skin tones, different hair textures, or length. Participants expressed that this was a challenge that made them feel stigmatized and invalidated in their Blackness. As such, some students felt like they fit in with neither the White students nor the Black students at their university. These findings are consistent with prior research, which highlights that cultural invalidations due to appearance happen often within the Black community, especially between women (Rockquemore, 2002). The current study extends this work by demonstrating that invalidations can be experienced not only by biracial individuals but by monoracial individuals as well. This may be particularly detrimental to Black undergraduate students’ well-being, given the importance of the peer network in more independently navigating new experiences in emerging adulthood (O’Connor et al., 2011; Ragelienė, 2016; Young et al., 2014) and the loss of supportive networks resulting from rejection and alienation by their same-race peers. More specifically, this could have implications for Black undergraduate students’ racial identity development as well as their mental health functioning (Yip, 2016). The consequences of cultural invalidations through colorist biases and stereotypes, primarily regarding physical characteristics such as skin tone, masked as “jokes,” harmfully impact Black undergraduate students and their identity within their community and can lead to feelings of depression and loneliness among peers (Green et al., 2023). Further, current findings are consistent with the prior research grounded in the minority stress theory, which highlights cultural invalidations as interpersonal stressors that impact the daily experience of Black individuals. Research suggests that repeated invalidating interactions, especially those occurring with same-race peers, may contribute to emotional dysregulation (Cardona et al., 2022; Ilagan et al., 2025).

Second, the current study emphasized the harmful consequences of Black individuals’ ascriptions to the misconception that there is a particular way to be Black (i.e., a monolithic perception of Blackness). Participants highlighted the internal struggle between being one’s authentic self and conforming to peer expectations of what to do or what not to do as a Black person. This included navigating racial stereotypes and expectations that were projected onto them. These findings relate to literature on “acting White,” in which individuals who do not conform to external expectations of what it means to be Black (e.g., speech patterns, style of dress, engaging in particular hobbies) are alienated by their community (Williams et al., 2019; Murray et al., 2012). Similar to the consequences of colorism, these experiences can result in individuals isolating themselves and experiencing negative mental health consequences (Green et al., 2023).

Green et al. (2023) found in their study of depressive symptoms and racial pride in biracial Black–White adolescents and emerging adults that experiencing cultural invalidations can lead to depressive symptoms. However, more pride in one’s biracial and Black identity was associated with fewer depressive symptoms. The current findings align with that study in that some participants who expressed a strong Black identity more easily dismissed cultural invalidations compared to individuals who did not express as strong a racial identity. In this society, it is important to be aware and actively fight against racial stereotypes and biases that negatively affect the Black community, regardless of one’s skin tone or lived cultural experience.

### 4.1. Limitations

The current study provides important insight into the within-group racial experiences of Black undergraduate students; however, it is not without limitations. First, findings from the current study emerged from data collected during a prior study that explored peer racial socialization among Black undergraduate students. As such, interview protocol questions were not tailored to gain an in-depth assessment of the experiences of cultural invalidations among this population. Additional research should be conducted to further explore the experiences of cultural invalidations with and among Black peers and the impacts of these invalidations on the lives of Black undergraduate students. Second, the current study provides insight into the experiences of Black undergraduate students at one university in a specific racial and political context. As such, to better understand the experiences and impacts of cultural invalidations across Black undergraduate students, it is important to examine this phenomenon in additional contexts. Lastly, the current study focuses on instances of colorism among Black students and their influence on identity development and sense of belonging. As this was the primary aim of the study, we did not examine potential influences of social class. We acknowledge, however, that aspects of colorism are historically intertwined with social class (Frazier, 1957). Therefore, future research should more explicitly examine how social class shapes experiences of colorism within Black communities.

### 4.2. Implications

Findings from the current study have important implications for future research and practice. First, future research should further examine how cultural invalidations perpetrated by in-group members impact Black undergraduate students’ racial identities as well as their mental health functioning. Given the importance of peers at this developmental stage, alienation and rejection from peers may lead to feelings of loneliness, hopelessness, and anxiety. Further, racial identity has been widely identified as a potential protective factor when Black individuals experience racial discrimination (Neblett et al., 2012; Yip et al., 2019). Rejection from individuals in one’s own racial group could result in individuals questioning their racial identity and may further leave them vulnerable to experiences of racial discrimination and its subsequent impacts. While additional research is necessary to better understand the implications of these experiences on Black undergraduate students’ racial identities and mental health functioning, findings from this study should inform clinical practice with Black undergraduate students. While assessing systems of support is a standard practice in the intake process of therapy, participants highlighted how systems that may be supportive (i.e., adaptive cultures) in some ways may also be significant sources of distress for Black undergraduate students. As such, additional attention should be given to assessing Black undergraduates’ peer relationships to better evaluate individuals’ available support systems and to aid in further development and maintenance of these sources of support. Practitioners should consider how experiences with same-race peers may lead to distress and subsequently impact an individual’s functioning in the areas of mental health and academic functioning as well.

Finally, addressing these challenges requires creating environments that promote positive racial and cultural identities and encouraging open discussions about all forms of racism and racial bias and their impacts. This includes dealing with the broad spectrum of racial teasing, from accusations of “acting White” to making derogatory remarks about someone’s cultural background or physical appearance (Horvat & O’Connor, 2006; Neal-Barnett et al., 2010). Education is crucial in raising peer awareness about the harm caused by racial teasing and the importance of supportive alliances (Suyemoto & Fox Tree, 2006). Through enhanced awareness, educational initiatives, and a dedication to building supportive, inclusive communities, individuals can heal from the scars of internalized racism and overcome feelings of inadequacy to develop a stronger, more positive self-identity, surrounded by peers who embrace diversity and mutual respect (Killen & Rutland, 2022).

## Data Availability

The original contributions presented in this study are included in the article. Further inquiries can be directed to the corresponding author.
